# Life-Threatening Upper Gastrointestinal Hemorrhage in Hemosuccus Pancreaticus: A Case Report

**DOI:** 10.7759/cureus.23934

**Published:** 2022-04-07

**Authors:** Amey Joshi, Jayashankar CA, Lakshmi Meghana Gada, Shruthi K.R., Kolli Chaitra

**Affiliations:** 1 General Medicine, Vydehi Institute of Medical Sciences and Research Centre, Bangalore, IND; 2 Internal Medicine, Vydehi Institute of Medical Sciences and Research Centre, Bangalore, IND; 3 Gastroenterology, Vagus Hospital, Bangalore, IND

**Keywords:** endovascular embolization, chronic pancreatitis, hypovolemic shock, pseudoaneurysm, hemosuccus pancreaticus

## Abstract

Hemosuccus pancreaticus (HP) is a rare cause of upper gastrointestinal bleeding caused by bleeding from the ampulla of Vater into the duodenum. HP most commonly results from a rupture of pseudoaneurysms secondary to chronic pancreatitis. The low incidence of HP and the wide spectrum of its clinical presentation poses diagnostic challenges. We present a case of a 39-year-old male with acute-on-chronic pancreatitis resulting in HP and obstructive jaundice due to pancreatic pseudocyst with secondary hematoma. This case highlights the rare occurrence of hypovolemic shock due to massive hemorrhage in HP and the successful management with prompt cardiovascular support and angiographic coil embolization of a bleeding pancreatic pseudoaneurysm.

## Introduction

Hemosuccus pancreaticus (HP), defined as bleeding from the ampulla of Vater into the duodenum through the pancreatic duct, is a rare cause of upper gastrointestinal (GI) bleeding [1]. While most commonly seen as a complication of chronic pancreatitis, this pathology can also be observed in a range of other conditions, including alpha-1 antitrypsin deficiency, pancreatic neoplasms, elastic tissue disorders, and vasculitis [2]. Although clinically characterized by a triad of abdominal pain, GI bleeding, and hyperamylasemia, HP can present with a broad spectrum of clinical features, including cardiovascular collapse [3]. Prompt identification of this pathology and timely intervention is paramount to ensure the patient's survival. Herein, we present a case of a young male with acute-on-chronic pancreatitis who developed hypovolemic shock secondary to HP.

## Case presentation

A 39-year-old male with a notable background of chronic alcoholism and a five-pack-year smoking history presented with complaints of intermittent epigastric pain of six-month duration. The patient described the pain to be dull-aching which was exacerbated by meals and radiated to the back. The patient also reported a one-month history of jaundice and dark-colored urine. The patient's past history was notable for a hospital admission one year ago for complaints of acute pain abdomen diagnosed as acute pancreatitis. Prior to the present admission, the patient was not known to be taking any prescription medications or noted to have any recent infection. 

Physical examination was noteworthy for pallor, icterus, and bilateral lower limb edema. The patient's abdominal examination revealed epigastric tenderness and hepatomegaly. Laboratory investigations revealed anemia, mild leucocytosis, elevated erythrocyte sedimentation rate (ESR), mildly elevated serum amylase (66 IU/l), and lipase (171 IU/l) levels, and hyperbilirubinemia (8.13 mg/dl) with a predominantly conjugated bilirubinaemia (4.88 mg/dl). Liver enzymes were within normal limits except for elevated alkaline phosphatase (ALP) of 333 IU/l (Table 1). Work-up for anemia revealed normal serum ferritin, transferrin saturation, vitamin B12, and folic acid levels. The stool tested for occult blood was negative. Urine chemistry revealed the presence of bilirubin and urobilinogen. The patient's serology also tested negative for Human Immunodeficiency Virus (HIV) by enzyme-linked immunosorbent assay (ELISA), Hepatitis B surface antigen (HBsAg), and Hepatitis C RNA. An abdominal ultrasound revealed features suggestive of acute-on-chronic pancreatitis with common bile duct (CBD) dilatation secondary to a benign stricture and cholelithiasis. Evidence of a well-defined hypoechoic lesion measuring 31 x 21 x 26 mm with echogenic contents and calcifications was also noted at the head of the pancreas. Upper gastrointestinal endoscopy was performed, which revealed features of oesophageal candidiasis. Endoscopic retrograde cholangiopancreatography (ERCP) was attempted; however, localized edema at the ampulla of Vater did not allow for the passage of scope. The patient was managed conservatively with fluid resuscitation, antibiotics, and antifungals, and was asked to review in two weeks duration.

**Table 1:** Blood parameters in the chronological day of hospital admission for the study case Hb: Hemoglobin, AST: Aspartate aminotransferase, ALT: Alanine transaminase, ALP: Alkaline phosphatase.

Laboratory parameter	Patient Value					Reference range
	Day 1	Day 14	Day 23	Day 26	Day 30	
Hb (g/dl)	9	9	4.9	-	8.8	13.2-16.6
Total count (µl)	11,600	12,000	21,000	-	6900	3400-9600
Platelet count (µl)	2.02	2.31	2.8	-	1.45	1.5-4.0
Blood urea (mg/dl)	15.1	8.2	7.7	-	-	6-24
Serum creatinine (mg/dl)	0.42	0.35	0.67	-	-	0.7-1.3
Serum Amylase (IU/l)	171	120	160	-	-	40-140
Serum Lipase (IU/l)	66	137	140	-	-	0-160
Total bilirubin (mg/dl)	8.13	14.76	3.72	2.57	-	0.2-1.0
Direct bilirubin (mg/dl)	4.88	9.14	2.18	1.32	-	0.1-0.3
Total protein (g/dl)	7	6.7	3.3	4.3	-	6.0-8.3
Albumin (mg/dl)	2.4	2	1.1	1.6	-	3.5-5.5
AST (IU/l)	59	56	119	58	-	8-48
ALT (IU/l)	64	44	42	39	-	7-55
ALP (IU/l)	333	260	76	92	-	40-129
Globulin (g/dl)	4.6	4.7	2.2	2.7	-	2.0-3.9
Prothrombin time (seconds)	20.9	24.7	-	-	19.6	11-13.5
International normalized ratio	1.78	2.12	-	-	1.67	<1.1
Serumsodium (mEq/l)	131.7	-	134.7	-	-	135-145
Serum potassium (mEq/l)	4.36	-	3.81	-	-	3.5-5.0
Serum calcium (mg/dl)	8	-	6.8	-	-	8.6-10.3

**Figure 1:**
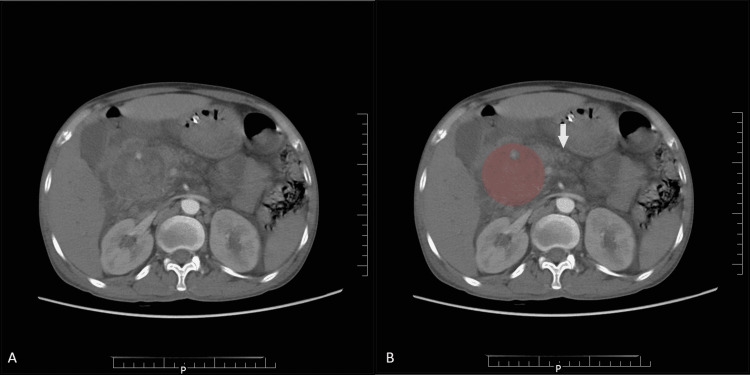
Contrast-enhanced CT axial sections of the abdomen CT: Computed Tomography Images A and B show pseudocyst of the pancreas measuring 55 x 54 mm (red in B) and hematoma (green in B) located posteriorly to the pancreas (arrow in B).

The patient presented to our emergency services two days (Day 14) later complaining of increasing abdominal pain. Laboratory investigations revealed a worsening of hyperbilirubinemia (14.76 mg/dl). Contrast-enhanced CT scan of the abdomen further revealed a pseudocyst of the pancreas (55 x 54 mm) with hematoma at the head of the pancreas, causing dilatation of the CBD, main pancreatic duct, and intrahepatic biliary radicles, and a pseudoaneurysm of the superior pancreaticoduodenal artery (Figure 1).

**Figure 2:**
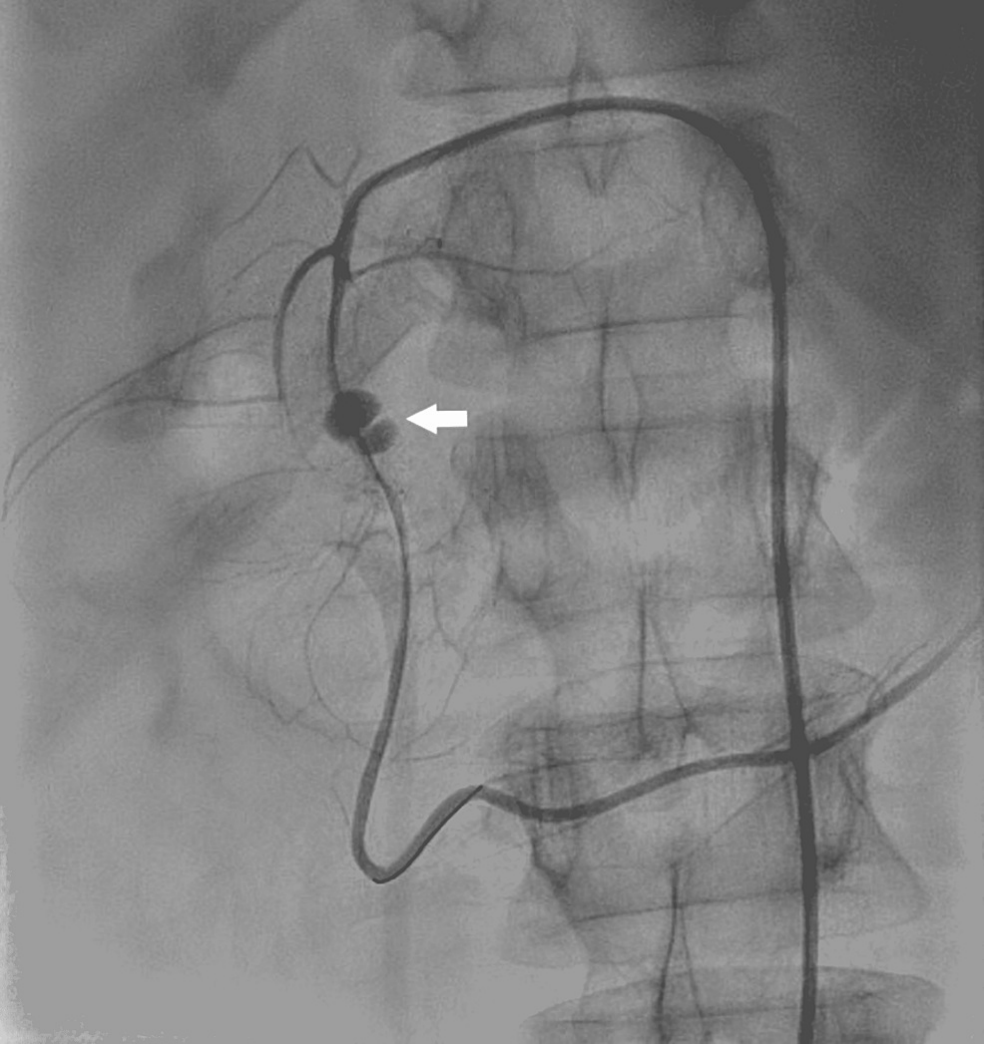
CT angiography via the right femoral route CT: Computed Tomography The arrow shows a superior pancreaticoduodenal artery pseudoaneurysm.

On day 23 of hospitalization, the patient reported a significant increase in the intensity of his abdominal pain. Tachycardia, tachypnoea, hypotension (mean arterial pressure {MAP} = 40 mmHg), abdominal guarding, and rigidity were noted. Suspecting the rupture of the pseudoaneurysm of the superior pancreaticoduodenal artery with secondary hypovolemic shock, a massive transfusion protocol and dopamine infusion were initiated, and Ryle's tube was inserted, which aspirated 1.5 L of frank blood. CT angiography confirmed the presence of the superior pancreaticoduodenal artery pseudoaneurysm with an active bleed into the pancreas (Figure 2).

**Figure 3:**
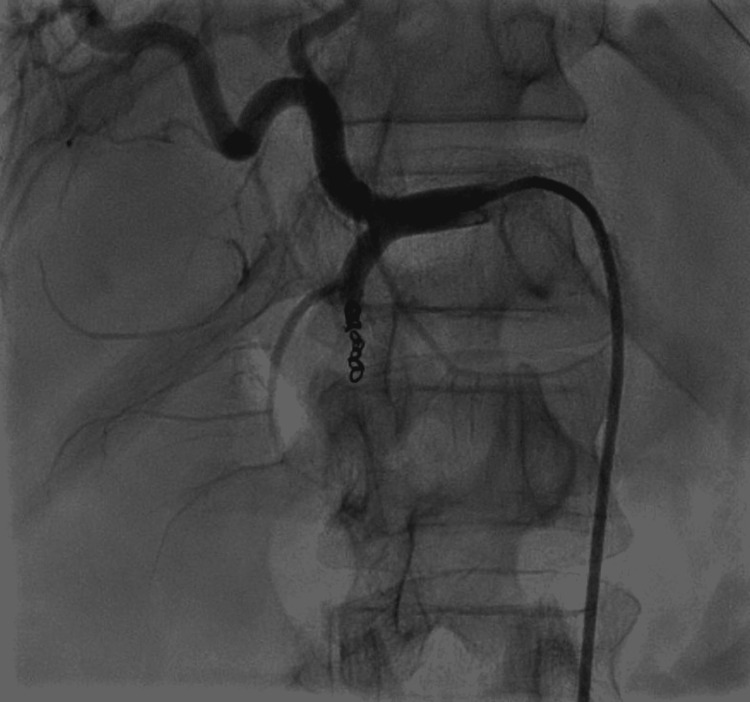
Coil embolization performed to the bleeding superior pancreaticoduodenal artery

The bleeding vessel was subsequently embolized using coil embolization (Figure 3). Repeat angiography showed a successful embolization and no further bleeding. Three units of fresh frozen plasma, three units of packed red blood cells, and four units of random donor platelets were transfused post-procedure. The patient's general condition improved subsequently, and he was discharged on close follow-up (Day 30). CT abdomen repeated one month later revealed a resolving pseudocyst and chronic calcific pancreatitis with the embolization coil in-situ.

## Discussion

Acute pancreatitis (AP), a state of localized destruction of the pancreas and systemic inflammatory response, is one of the leading causes of worldwide gastrointestinal-related hospitalizations [4]. Recurring AP and the interplay of genetic and environmental factors can lead to the development of chronic pancreatitis (CP) in a small fraction of patients. The sequelae of CP, such as splenic venous thrombosis, pseudocysts, pseudoaneurysms, and endocrine and exocrine insufficiencies, pose challenges to practitioners worldwide [5]. A pancreatic pseudoaneurysm, an erosion of a pancreatic or peripancreatic artery into an existing pancreatic pseudocyst, is an uncommon clinical entity that affects nearly 10% of patients with CP [6]. The rupture of this pseudoaneurysm into the pancreatic duct can result in upper GI bleeds having detrimental consequences and high mortality. This phenomenon, called hemosuccus pancreaticus, was first reported in 1931 and later defined in 1970 [7]. Herein, we report a case of a young male who presented with acute-on-chronic pancreatitis and consequential hypovolemic shock secondary to hemosuccus pancreaticus. This was successfully managed by promptly identifying the pathology and angiographic coiling and embolizing the diseased superior pancreaticoduodenal artery. 

Nearly 80% of all HP can be attributed to acute or chronic inflammatory processes of the pancreas. This may occur due to the necrosis and disruption of the vessel wall or, more commonly, the rupture of pseudoaneurysm (10%) [2]. The most frequently involved arteries in this pathology include the splenic artery (60-65%), gastroduodenal artery (20-25%), and pancreaticoduodenal artery (10-15%). When these diseased arteries or the pseudoaneurysms open into the pancreatic duct, they drain into the second part of the duodenum through the ampulla of Vater, resulting in an upper GI bleed [2]. The symptoms may range from melena to catastrophic hypovolemic shock. HP due to a directly communicating fistula between a pseudoaneurysm and duodenum has also been reported [8]. Other reported causes of HP include primary and metastatic pancreatic neoplasms, vascular anomalies like polyarteritis nodosa, α-1 antitrypsin deficiency, fibromuscular dysplasia of the arterial wall, and elastic tissue defects like Marfan's syndrome, and Ehlers-Danlos [9-11]. Few cases of HP secondary to diagnostic and therapeutic endoscopic procedures like endoscopic ultrasonography and ultrasound-guided drainage of pancreatic cysts have been reported [2]. 

The clinical features of HP include a triad of symptoms; epigastric pain, GI bleeding, and hyperamylasemia (raised levels of serum amylase) [3]. HP has often been referred to as psuedohemobilia because of its extrahepatobiliary origin [12]. On the other hand, hemobilia is characterized by a triad of symptoms very similar to that of HP with one notable difference: the exclusion of hyperamylasemia and inclusion of jaundice [2]. Surprisingly, our patient had significant conjugated hyperbilirubinaemia due to biliary stasis. This may be due to the extra-biliary compression of the CBD by the pancreatic pseudocyst. The presence of hyperbilirubinemia, therefore, should not exclude the diagnosis of HP.

In HP, patients often present with intermittent abdominal pain and melena. This may be due to the formation and dissolution of clots in the pancreatic duct or pseudocyst. Although bleeding into the gut due to HP is more commonly not severe, a few cases of massive GI hemorrhages leading to hemodynamic instability have been reported [13,14]. Similarly, an acute hemodynamic decompensation was observed during the patient's hospital stay due to a massive GI hemorrhage that led to hypovolemic shock requiring vasopressor support and repeated blood transfusions. This clinical picture may result from the dissolution or dislodgement of a clot that may have initially been present at the ampulla of Vater. The resolution of the hematoma and resolving pseudocyst may have subsequently reduced the extra-biliary mass effect on the CBD resulting in the improvement of the patient’s hyperbilirubinemia.

Diagnosing HP may be challenging due to its rarity, varying presentation, and complex anatomical site. Diagnostic modalities including esophagoduodenoscopy, contrast-enhanced CT, angiography, and ERCP are required to diagnose HP [7]. Early endoscopy may help differentiate HP from other possible causes of upper GI bleed, including oesophageal and gastric varices and peptic ulcers [7]. The identification of active ooze or bleeding from the ampulla of Vater should raise suspicion of hemobilia or HP [2]. In the present case, although esophagoduodenoscopy was done during the patient's initial presentation, we did not observe an active bleed from the ampulla. This reiterates that although upper GI endoscopy may be the initial choice of investigation in upper GI bleeds, it fails to detect bleeding via papilla in over 70% of patients [15]. Although ERCP was warranted in the present case, we could not successfully perform this procedure due to localized oedema at the papilla. Angiography can detect HP with a sensitivity of 96% and current angiographic protocols focus on the detection of active bleeding and pseudoaneurysm formation [16]. Furthermore, the localization of active bleeding can allow prompt transcatheter embolization to achieve hemostasis [16]. MRI angiography has also been proposed as an additional reliable diagnostic tool for the diagnosis of HP. This may provide better visualization of the ampulla and detect trace blood in the pancreatic duct with the added benefit of no radiation exposure [17]. 

Due to the high mortality of HP when managed supportively (90%) [18], prompt management using an interventional radiological approach or surgical approach must be considered [7]. The interventional radiological approach may be considered in hemodynamically stable patients diagnosed with HP with angiography. Balloon tamponade, coil embolization, embolization using prosthetic material, and stents are generally employed. Recurrence of bleeding has been reported in 30% of patients undergoing embolization. The mortality rate in patients undergoing embolization has been documented to vary from 6% to 33% [2,7]. Surgical approaches include intracystic suture ligation with proximal and/or distal arterial ligation, partial pancreatectomy, splenectomy with distal pancreatectomy, total or partial gastrectomy or hemicolectomy, pancreaticoduodenectomy, and necrosectomy [1]. Surgical success rates vary from 70 to 85%, with a 10-50% mortality risk. The chances of rebleeding associated with a surgical approach are meager (0-5%) [2]. In the present case, we used coil embolization to stop the bleeding from the superior pancreaticoduodenal artery pseudoaneurysm by providing prompt cardiorespiratory support and ensuring hemodynamic stability before the procedure. 

Follow-up evaluation of patients with acute pancreatitis should include assessments of pancreatic exocrine and endocrine function, recurrent cholangitis, and development of infected fluid collections. Although no evidence-based guidelines are currently available regarding out-patient follow-up care, patients should generally be asked to review within 7-10 days from hospital discharge. It is important to note that if the pancreatitis was moderate to severe and associated with peripancreatic fluid collections, imaging studies during the follow-up period are indicated to exclude pseudocyst formation [19]. CT scan to detect pseudoaneurysm, chronic pancreatitis, or neoplasia is a preferred first-line investigation during follow-up care. Magnetic Resonance Cholangio-pancreatography (MRCP) is indicated if CT scans are non-diagnostic and may help identify strictures, developmental abnormalities, or evidence of chronic pancreatitis. Endoscopic ultrasonography can also aid in diagnostics for detecting biliary sludge, microlithiasis, and periampullary lesions [19,20].

## Conclusions

HP is a condition that has a broad spectrum of clinical presentations ranging from intermittent epigastric pain and melena to catastrophic GI haemorrhage. The varied presentation of HP and the limited literature evidence due to its rarity make it challenging to diagnose. This condition, if not identified early, can result in hemodynamic collapse. The presence of conjugated hyperbilirubinemia in HP must raise suspicion of the possible mass effect of an underlying pancreatic pseudocyst or pseudoaneurysm on the CBD. Diagnostic modalities include contrast-enhanced CT scans, endoscopic procedures like esophagoduodenoscopy and ERCP, and radiological procedures like angiography. Therapeutic intervention through an interventional radiological approach using coil embolization is safe and effective in hemodynamically stable patients with HP. 
